# Schizencephaly—diagnostics and clinical dilemmas

**DOI:** 10.1007/s00381-015-2638-1

**Published:** 2015-02-18

**Authors:** Agata Halabuda, Lukasz Klasa, Stanislaw Kwiatkowski, Lukasz Wyrobek, Olga Milczarek, Aleksandra Gergont

**Affiliations:** 1 Department of Children’s Neurosurgery, Polish–American Institute of Pediatrics/Jagiellonian University, Kraków, Poland; 2 Department of Children’s Neurology, Polish–American Institute of Pediatrics /Jagiellonian University, Kraków, Poland; 3 Department of Radiology, Polish–American Institute of Pediatrics/Jagiellonian University, 265 Wielicka St., 30-663 Kraków, Poland

**Keywords:** Schizencephaly, EMX2, Hydrocephalus, Arachnoid cyst

## Abstract

**Background:**

Schizencephaly is an uncommon congenital disorder of cerebral cortical development. The defect is characterized by the presence of a cleft in the brain extending from the surface of the pia mater to the cerebral ventricles. The margins of the cleft are lined with heterotropic, dysplastic gray matter. The causes of schizencephaly are heterogeneous and can include teratogens, prenatal infection, maternal trauma, or EMX2 mutations.

**Method:**

In the present paper, the authors described difficulties in employing diagnostic imaging in differentiating between type II (open-lip) schizencephaly and much more common intracranial fluid spaces of a different origin (arachnoid cysts and hydrocephalus).

**Result:**

In all the three cases, the treatment consisted in implantation of a shunt system; nevertheless, it should be emphasized that a surgical intervention in the third presented case (type II schizencephaly) aimed at relieving the symptoms of intracranial hypertension—a directly life-threatening condition—since shunting is not a method of treating schizencephaly itself.

**Conclusions:**

Although proper interpretation of the character of intracranial fluid spaces is of significance for further therapeutic management, yet, the key decision as to the surgical intervention is made based on clinical presentation, predominantly on symptoms of intracranial hypertension.

## Introduction

Schizencephaly is a rare congenital CNS malformation belonging to the group of cell migration defects, which develop between 2 and 5 months of gestation. The defect is characterized by the presence of a cleft in the brain extending from the surface of the pia mater to the cerebral ventricles. The margins of the cleft are lined with heterotrophic, dysplastic gray matter. The most common location of the anomaly is the frontal lobe and the region of the lateral sulcus [1–4].

The malformation was first described by Wilmarth in 1887, while in 1946, Yakovlev and Wadsworth, basing their observations on five patients with neurological deficits and brain deformations, described two types of schizencephaly—type I (closed-lip), a form that does not communicate with the ventricular system, and type II (open-lip) that presents with communication with the ventricular system [5].

The incidence of the defect is reported as 1/1650 patients with epileptic seizures and/or psychomotor retardation or 1.54/100,000 births [6–8]. This malformation may be unilateral or bilateral, with bilateral schizencephaly being slightly more common. In case of bilateral schizencephaly, 60 % of cases are bilaterally open, 20 % of cases are unilaterally open, and 20 % of cases are bilaterally closed [4, 9, 10]. In case of unilateral schizencephaly, 60 % are open [4, 9, 10].

## Etiology

The etiopathogenesis of schizencephaly has not been fully elucidated. One concept postulates the effect of external factors, which damage the developing fetal brain. In keeping with this idea, the cause of schizencephaly is middle cerebral artery stroke in consequence of an inflammatory process occurring in utero, e.g., as an effect of cytomegalovirus infection. The hypothesis is supported by the fact that the majority of schizencephaly clefts are seen in the lateral sulcus region and thus in the middle cerebral artery vasculature [11–14].

Other risk factors of schizencephaly development include also maternal young age (below 20 years of life), no prenatal medical care (especially in the first trimester of pregnancy), and abuse of alcohol and narcotic substances as well as using some medications by pregnant women (e.g., warfarin). Cases of bilateral schizencephaly corpus callosum hypoplasia have been described in children with fetal alcohol syndrome (FAS) [5]. Another theory points to genetic factors as the cause of schizencephaly development, although numerous authors cast doubt at the concept [8].

In keeping with the above theory, children with schizencephaly have a heterozygotic mutation of the EMX2 gene, which is a regulating gene for structural development of the prosencephalon [15]. The advocates of the theory are of the opinion that other malformations, which may present concomitantly with schizencephaly, such as polymicrogyria, agenesis of the septum pellucidum and/or corpus callosum, optic nerve atrophy, arachnoid cysts, and cerebellar anomalies, provide an argument in support of the genetic background of schizencephaly [16]. Such background is also supported by schizencephaly described in the extremely rare Vici syndrome (corpus callosum agenesis, albinism, immune deficiency, cardiomyopathy), which develops in consequence of the EPG5 gene mutation [5]. Nevertheless, the majority of authors currently believe that there is insufficient evidence that would confirm the validity of the theory, since the EMX2 gene mutation is noted only in some children with schizencephaly. Thus, it appears that even if in some part of cases, genetic background is involved in schizencephaly development; such a cause is very rare [8].

## Diagnostic imaging

The method of choice in diagnostic imaging of schizencephaly is MRI. CT is also useful, but to a lesser degree, since it provides poorer images of the gray matter, which are the key factor in differentiating between the malformation and other fluid-associated CNS abnormalities. Schizencephaly may be also diagnosed in prenatal or postnatal ultrasonography, but this is true for type II (open-lip) only.

Medical imaging shows schizencephaly as a linear cleft lined with heterotrophic gray matter and extending from the cortical surface to the ventricular system. The gray matter within the cleft is dysplastic (polymicrogyria) [15, 17]. Clusters of abnormal gray matter may be observed not only within the cleft, but also in its vicinity, without any clear communication with the cleft. In case of unilateral schizencephaly, dysplastic gray matter may be located in the contralateral hemisphere in the same or similar location, forming the so-called mirror focus. Dysplastic gray matter may constitute an epileptogenic zone [1–3, 18].

Other concomitant pathologies that may be observed in patients with schizencephaly include hydrocephalus (in approximately 30 % of cases and almost exclusively in the open-type schizencephaly) [7] and the above-listed agenesis of the septum pellucidum and/or corpus callosum, optic nerve atrophy, arachnoid cysts, and cerebellar malformations [11].

## Clinical presentation

Schizencephaly, especially its type II, is a severe irreversible CNS malformation, which is manifested by epilepsy, often refractory, and varying degrees of paralysis—hemiparesis in case of unilateral schizencephaly and quadriparesis in bilateral schizencephaly. The malformation is additionally associated with mental retardation and, in case of severe forms of schizencephaly located in the frontal lobe or in the lateral sulcus region, with varied forms of characteropathy. Type I schizencephaly (closed-lip) is characterized by a markedly milder course. The defect may be asymptomatic or diagnosed only in adult patients. It presents with epileptic seizures and mild motor deficits [11, 19, 20]. EEG imaging has demonstrated that the epileptogenic zone is the dysplastic cortex, which—as it has been mentioned before—may be situated not only within the cleft, but also in its vicinity and in the contralateral hemisphere [15, 21].

As a rule, therapeutic management of both types of schizencephaly is conservative and predominantly consists in rehabilitation of motor deficits and mental retardation and treatment of epilepsy. Surgical treatment is undertaken only in some cases with concomitant hydrocephaly or intracranial hypertension [22, 23].

## Case presentation

### Case 1

**Figs. 1 and 2:**
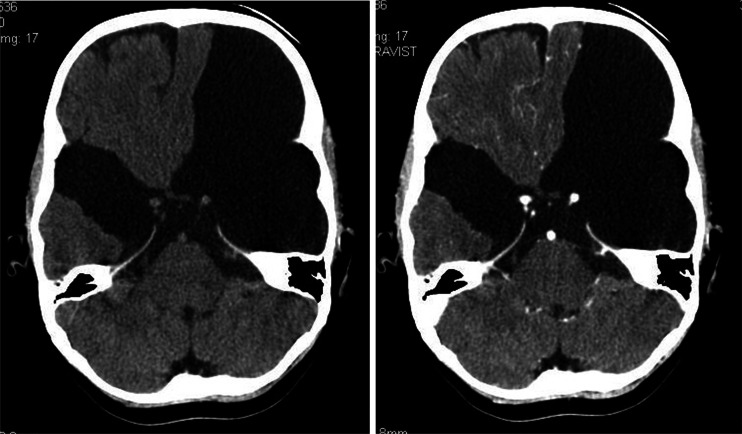
Arachnoid cysts of the lateral sulcus, the larger one being situated on the left side, a minimal mass effect. Nonenhanced CT (Fig. 1) contrast-enhanced CT (Fig. 2)

A 3-year-old boy admitted to the University Children’s Hospital of Krakow following the first epileptic seizure. The patient complained of headaches and vomiting. Based on clinical presentation and medical history, intracranial hypertension was suspected. CT scan demonstrated bilateral fluid-filled spaces in the middle cranial fossa (the larger being situated on the left side), with hypotrophy of the adjacent cerebral structures. In addition, CT showed a minimal mass effect manifested as displacement of the longitudinal fissure of the brain to the right and compression and stenosis of the left lateral cerebral ventricle, as well as mild thinning of the squama of the temporal bone at the level of the malformation (Figs. 1 and 2).

In differential diagnosis, consideration was given to the open type of bilateral schizencephaly or bilateral arachnoid cysts of the lateral sulcus, with type III on the left and type II on the right side (Galassi classification).

The CT scan favored the arachnoid cyst (the mass effect, discrete thinning of the squama of the temporal bone), yet schizencephaly could not have been ruled out based on a single CT scan and medical history of the patient. The scan was performed as an emergency procedure in a patient with severe and growing in intensity symptoms of intracranial hypertension, what did not allow for extensive, time-consuming diagnostic management.

**Fig. 3:**
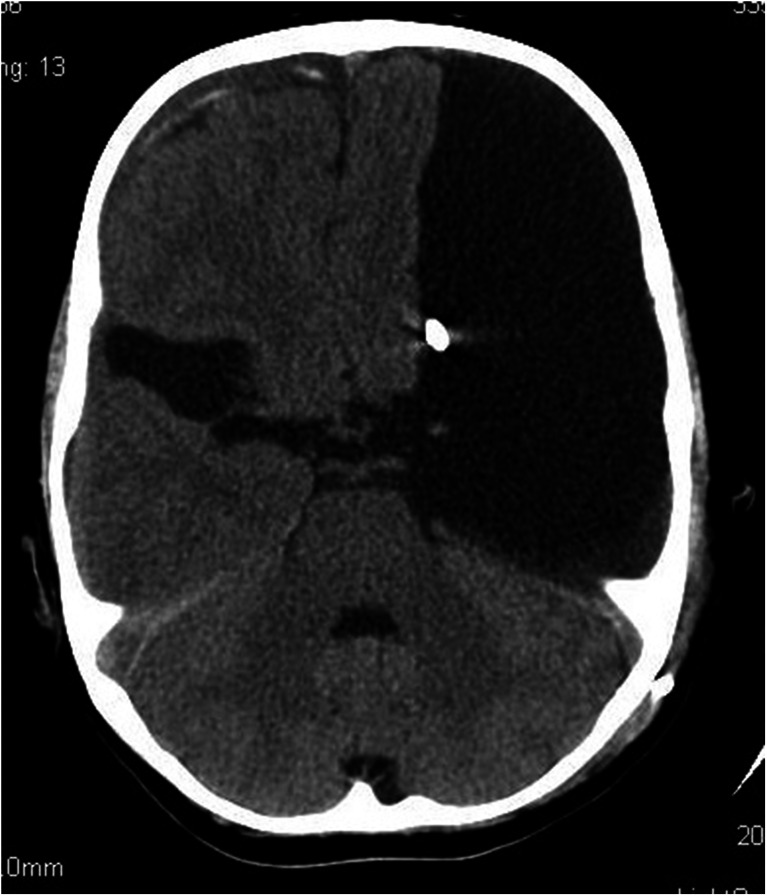
Status after implantation of a cysto-peritoneal shunt on the left side, a minimal mass effect, flat paracerebral hematoma hygromas in the vicinity of both frontal lobes, mild cerebral edema demonstrated as cerebral sulci, and fissure obliteration. Nonenhanced CT scan

**Fig. 4:**
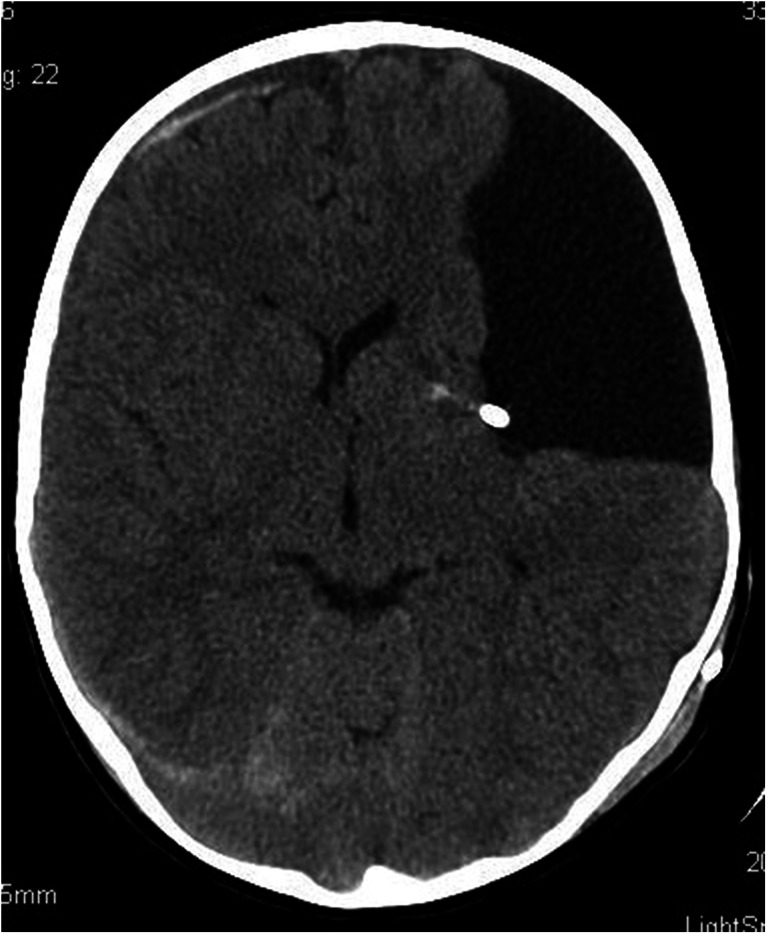
The photo presents the same scan as in Fig. 3, but a different cross section. A small subdural hygro-hematoma in the right frontal region. A blood trace along the tentorium of the cerebellum. A mass effect seen as minimal displacement of the lateral fissure of the brain to the right, with mild compression of the frontal corn of the left lateral ventricle. Nonenhanced CT scan

A decision was made on a surgical intervention consisting in implantation of a cysto-peritoneal shunt on the left side. Following shunting, the patient developed complications consisting in small intracerebral and paracerebral hematomas (Figs. 3 and 4).

**Fig. 5:**
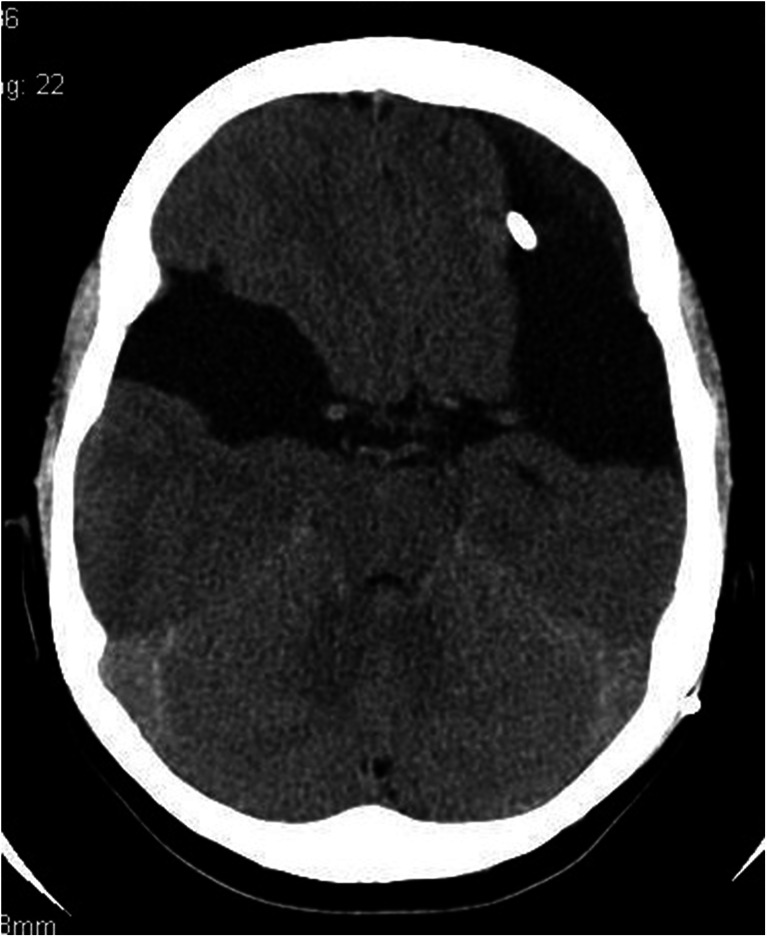
A follow-up scan, 3 years after shunt implantation: considerable regression of the bilateral arachnoid cysts in the middle cranial fossa. In the left frontal region, a narrow chronic subdural hygro-hematoma is shown, approximately 7 mm wide. Nonenhanced CT scan

In subsequent follow-up scans, gradual resorption of hemorrhagic lesions was seen. The fluid-filled spaces were still present; albeit markedly smaller as compared to the initial scan, discrete asymmetry of the lateral ventricles persisted, but no displacement of the lateral fissure of the brain was detected. The patient improved clinically (Fig. 5).

Following implantation of a drain system and clinical improvement of the patient, a MRI scan of the head was performed, which ultimately confirmed that the fluid-filled spaces in the two middle cranial fossae corresponded to arachnoid cysts.

### Case 2

A 1-month-old girl was admitted to the University Children’s Hospital of Krakow due to suspected intracranial hypertension. She had been previously diagnosed and treated elsewhere due to epilepsy and extensive bilateral intracranial fluid-filled spaces. As it followed from her medical history, the mother had had a toxoplasma infection in pregnancy.

**Fig. 6:**
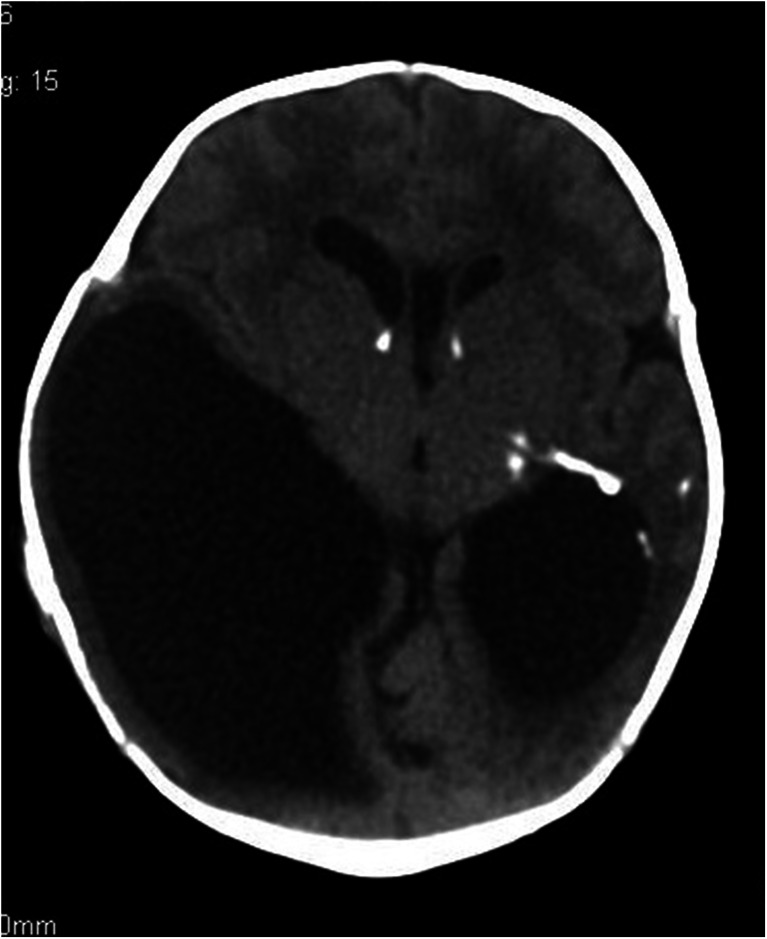
Massive bilateral distension of the occipital and temporal horns of the lateral ventricles of the brain. Periventricular calcifications. Incomplete brain myelination. Nonenhanced CT scan

A CT scan performed immediately upon admission demonstrated bilateral extensive fluid-filled spaces in the temporal and occipital lobes, which corresponded to markedly distended temporal and occipital horns of the lateral ventricles of the brain. Additionally, the scan showed agenesis of the corpus callosum and periventricular calcifications (Fig. 6).

**Fig. 7:**
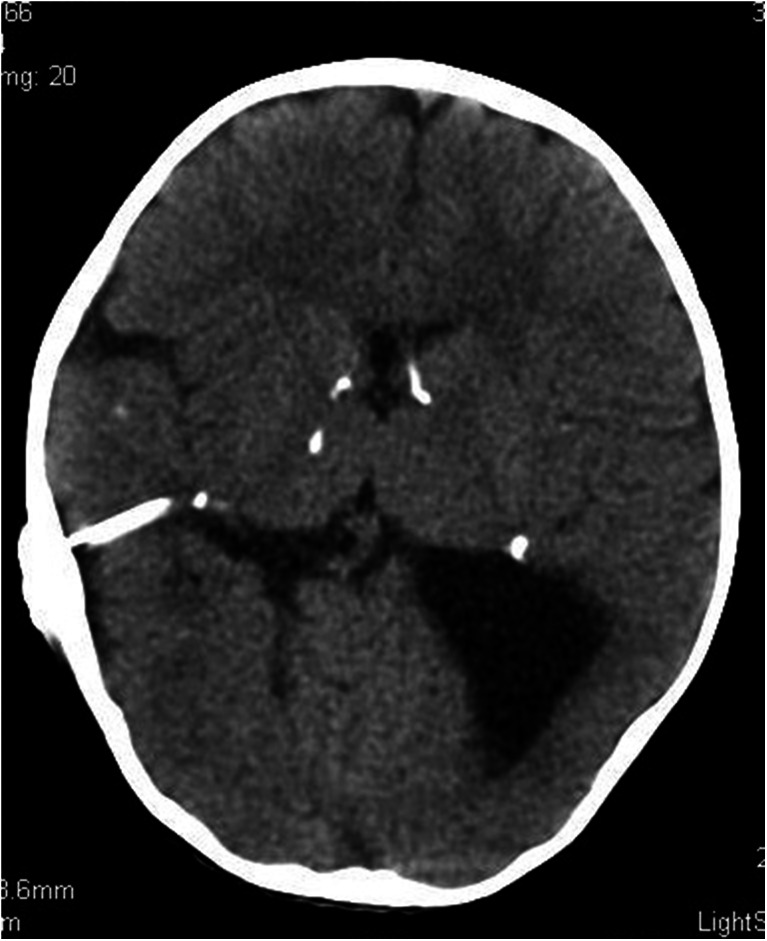
The scan was performed 7 months following ventriculoperitoneal shunting. Marked supratentorial hydrocephaly regression. Mildly distended occipital and temporal horns of the left lateral ventricle. Nonenhanced CT scan

The child was treated surgically by implanting a 70 mm H_2_O fixed-pressure ventriculoperitoneal shunt on the right side. No postoperative complications were noted (Fig. 7).

### Case 3

**Figs. 8 and 9:**
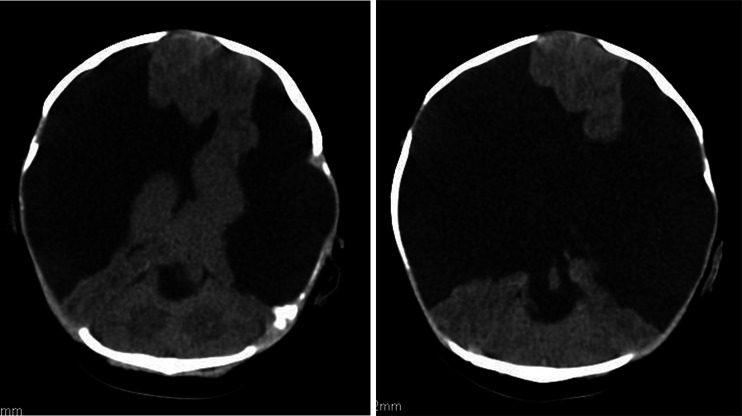
Bilateral open-type schizencephaly. The compressed sulci and longitudinal fissure of the brain represent signs of intracranial hypertension. Nonenhanced CT scan

A 1-month-old girl admitted to the Cracow University Children’s Hospital of Krakow with suspected intracranial hypertension and diagnosed epilepsy. A CT scan demonstrated bilateral fluid-filled spaces in the transverse fissure of the brain—bilateral open schizencephaly (Figs. 8 and 9).

**Fig. 10:**
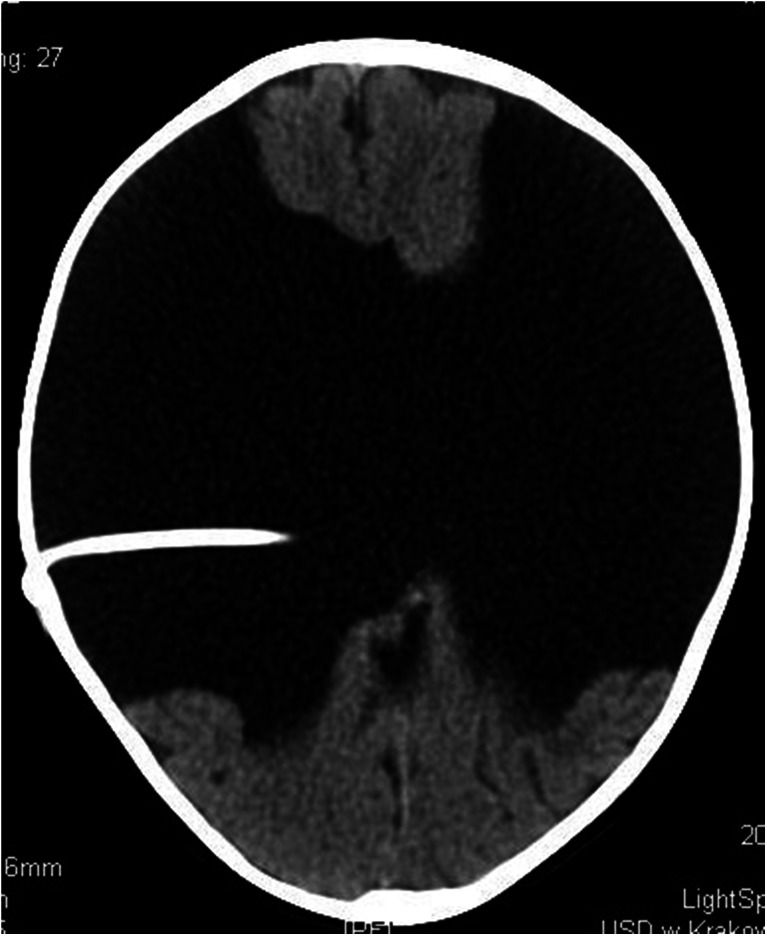
A follow-up scan 8 months following shunting. Persistent supratentorial fluid-filled spaces of a comparable size. The longitudinal fissure of the brain and brain sulci are seen more clearly as compared to the preoperative scan, what indicates decreased intracranial hypertension. Nonenhanced CT scan

In view of the clinical symptoms of intracranial hypertension, the child was implanted a cysto-ventriculoperitoneal shunt (Fig. 10).

Her clinical status improved. The girl is followed up by a neurologist due to refractory epilepsy.

## Discussion

A particular problem in differentia diagnosis is posed by open-type schizencephaly, which in medical imaging is seen as a fluid-filled space. Inappropriate interpretation of the image may result in inappropriate further management. In cases when imaging studies demonstrate intracranial fluid-filled spaces, especially when they are situated in the middle cranial fossa, differential diagnosis needs to take into consideration several pathologies. In the majority of cases, in this location, arachnoid cysts are seen. A very rare congenital pathology is schizencephaly. Differentiation between these two malformations poses a significant diagnostic problem. Inasmuch as therapeutic management of symptomatic forms of arachnoid cysts consists in a surgical intervention, in case of schizencephaly, the value of the method is doubtful and the modality is additionally associated with consequences in the form of postoperative complications.

Surgical management of an arachnoid cyst lies in creating communication between the cyst and physiological brain cisterns (fenestration) or in implanting a shunt system (currently considerably less common and performed mainly in case fenestration fails or the patient is disqualified from the method). Both methods are associated with a risk of such complications as postoperative bleeding, distension of the paracerebral fluid spaces forming subdural hygromas or hygro-hematomas, empyemas, meningitis, and hydrocephalus. In case when shunting is employed as the therapeutic modality (with the complication rate of approximately 50 %, the patient may additionally develop such complications as shunt system impatency, drain pullage and detachment, and ascites. If drainage is maintained for longer periods, complications may also include endocarditis and renal failure—shunt nephritis [23].

The most differentiating important element in imaging studies is the presence of heterotrophic gray matter that lines the margins of the cleft in case of schizencephaly and absence of such a lining in arachnoid cysts or fluid-filled spaces with other background. Moreover, arachnoid cysts may cause a mass effect manifested as displacement of the longitudinal fissure of the brain, compression of the ventricular system, or local obliteration of brain sulci and fissures, what is not evident in schizencephaly. In rare instances of arachnoid cysts, thinning and bulging of cranial bones are seen in areas where they adhere to the cyst.

Other disease entities that should be also considered in differential diagnosis of congenital arachnoid cysts and schizencephaly are acquired cysts (post-traumatic, postoperative, post-hemorrhage, or postictal). In such cases, a MRI scan shows areas of gliosis, i.e., glial scars, surrounding the fluid-filled lesions; medical history is also of assistance. Open-type schizencephaly should be also differentiated from hydrocephalus (a case discussed in the present paper) and from holoprosencephaly.

Three presented above cases of extensive intracranial fluid-filled spaces were qualified for surgical treatment in view of clinical symptoms of acute intracranial hypertension. Inasmuch as surgical treatment of arachnoid cysts and hydrocephalus is a commonly employed and accepted therapeutic modality, implantation of a shunt system in the third presented case deserves a separate discussion. In this case, the employed modality addressed acute intracranial hypertension rather than schizencephaly itself. The effect of the treatment was a resolution of symptoms of intracranial hypertension, which is a direct life-threatening condition and requires a prompt intervention.

On the other hand, schizencephaly itself and its associated symptoms are treated conservatively.

## References

[1] Bansal N, Maini B, Bhardwaj AK (2012). Schizencephaly of open and closed lip in the same patient: an extremely rare occurrence. J Pediatr Neurosci.

[2] Barkovich AJ, Kjos BO (1992). Schizencephaly: correlation of clinical findings with MR characteristics. AJNR.

[3] Barkovich AJ, Kuzniecky RI, Jackson GD (2001). Classification system for malformations of cortical development. Neurology.

[4] Osborn AG Salzman, KL, Barkovich AJ (2010) Schizencephaly. In Osborn Diagnostic Imaging Brain 2nd ed. Amirsys Publishing, Inc. Chapter 1 pp77-80

[5] Dies KA, Bodell A, Hisama FM (2013). Schizencephaly: association with young maternal age, alcohol use, and lack of prenatal care. J Child Neurol.

[6] Curry CJ, Lammer EJ, Nelson V (2005). Schizencephaly: heterogeneous etiologies in a population of 4 million California births. Am J Med Genet.

[7] Kopyta I, Jamroz E, Marszał E (2006). Schizencephaly—clinical and radiological presentation in patients at developmental age. Wiad Lek.

[8] Merello E, Swanson E, De Marco P (2008). No major role for the EMX2 gene in schizencephaly. Am J Med Genet A.

[9] Klimczak A, Mandera M (2007) Hydrocephalus in congenital defects of central nervous system. In Zakrzewski K (ed) Hydrocephalus and other disturbances of cerebro-spinal fluid circulation in children, 1st ed. Wydawnictwo Czelej Sp. Z o. o. pp 105-110

[10] Donelly LF et al (2005) Schizencephaly. In Donelly LF (ed) Diagnostic Imaging Pediatrics 1st ed. Amirsys Publishing, Inc. Chapter 7 pp 34-37

[11] Denis D, Chateil JF, Brun M (2000). Schizencephaly: clinical and imaging features in 30 infantile cases. Brain Dev.

[12] Fernandez-Bouzas A, Harmony T, Santiago-Rodriguez E (2008). Schizencephaly with occlusion or absence of middle cerebral artery. Neuroradiology.

[13] Hung PC, Wang HS, Yeh YS (1996). Coexistence of schizencephaly and intracranial arteriovenous malformation in an infant. AJNR.

[14] Iannetti P, Nigro G, Spalice A (1998). Cytomegalovirus infection and schizencephaly: case report. Ann Neurol.

[15] Granata T, Freri E, Caccia C (2005). Schizencephaly: clinical spectrum, epilepsy, and pathogenesis. J Child Neurol.

[16] Kuban KC, Teele RL, Wallman J (1989). Septo-optic dysplasia-schizencephaly. Pediatr Radiol.

[17] Caraballo RH, Cersósimo RO, Fejerman N (2004). Unilateral closed-lip schizencephaly and epilepsy: comparison with case of unilateral polymicrogyria. Brain Dev.

[18] Barkovich AJ, Norman D (1988). MR imaging of schizencephaly. AJNR.

[19] Hayashi N, Tsutsumi Y, Barkovich AJ (2002). Morphological features and associated anomalies of schizencephaly in the clinical population: detailed analysis of MR images. Neuroradiology.

[20] Chen H (2006) Schizencephaly. In Atlas of Genetic Diagnosis and Counseling 1st ed. Humana Press pp867-869

[21] Jankszy J, Ebner A, Kruse B (2003). Functional organization of the brain with malformation of cortical development. Ann Neurol.

[22] Inoue R, Isono M, Kamida T (2002). A case of schizencephaly with subdural fluid collection in a neonate. Child’s Nerv Syst.

[23] Kwiatkowski S (2007) Complications in shunting. In: Zakrzewski K (ed) Hydrocephalus and other disturbances of cerebro-spinal fluid circulation in children, 1st edn. Wydawnictwo Czelej Sp. Z o. o. pp 67-79

